# Synthesis and Study of the Properties of Dual-Substituted Amphiphilic Low-Molecular-Weight Chitosan Derivatives

**DOI:** 10.3390/polym18212649

**Published:** 2026-10-26

**Authors:** Balzhima Shagdarova, Nadezhda Vasileva, Alexey Lunkov

**Affiliations:** Research Center of Biotechnology of the Russian Academy of Sciences, Institute of Bioengineering, Moscow 119071, Russia; v4snadezhda@yandex.ru (N.V.); fwnf1994@gmail.com (A.L.)

**Keywords:** chitosan, hydrophobic chitosan derivatives, quaternized chitosan, derivatives with double substituents, self-assembly, cytotoxicity, drug delivery

## Abstract

A series of low-molecular-weight chitosan derivatives with varying degrees of hydrophobic and cationic substitution was synthesized using a reductive alkylation method. At the first stage, a hydrophobic C14-alkyloxybenzyl moiety was introduced into the macromolecule; at the second stage, a cationic quaternary ammonium substituent was introduced. The structure and degree of substitution of the resulting compounds were confirmed by ^1^H NMR and IR spectroscopy. The actual degrees of substitution ranged from 0.5 to 4.1% for the hydrophobic substituent and 6 to 18% for the cationic substituent. We investigated the pH-dependent aggregation of the derivatives, the sizes and ζ-potential of the nanoaggregates in acidic and physiological media (pH 7.4), as well as their cytotoxic activity against the SH-SY5Y, BT474, and Skov3 cell lines. It was shown that the introduction of hydrophobic moieties induces self-association of macromolecules, whereas cationic groups increase hydrophilicity and ensure that the aggregates retain a positive charge at physiological pH. Turbidimetric titration and dynamic light scattering revealed a nonlinear dependence of colloidal properties on the ratio of hydrophobic to cationic substituents. The most stable systems were formed at moderate hydrophobicity and a low degree of quaternization, whereas an increase in the content of hydrophobic groups enhanced aggregation. Cytotoxic activity of the derivatives depended on their substitution pattern, polymer concentration, and cell line. Although higher degrees of substitution are associated with increased cytotoxicity, this dependence is not universal and depends on the balance between the two types of substituents. The results obtained demonstrate the potential of the synthesized amphiphilic chitosan derivatives as a platform for developing nanoscale delivery systems for biologically active substances.

## 1. Introduction

Chitosan is a linear polysaccharide whose chain consists of a random sequence of β-(1→4)-linked units of D-glucosamine and *N*-acetyl-D-glucosamine. The presence of free primary amino groups distinguishes chitosan from most natural polysaccharides and allows for selective chemical *N*-modification of the polysaccharide units. The ability to vary the molecular weight, degree of acetylation, degree of substitution, and structure of the substituent makes chitosan a convenient platform for developing functional polymeric materials with tunable physicochemical properties [1,2,3]. In our study, we used low-molecular-weight chitosan (LMW-chitosan), since molecular weight affects the properties of chitosan solutions, such as viscosity and the nature of association [4,5,6]. LMW chitosan, obtained by acid hydrolysis, has a higher degree of deacetylation (96%), which provides a greater number of available amino groups (NH_2_ groups). It also has significantly lower solution viscosity, which contributes to a more uniform course of chemical modification. Thus, LMW chitosan is a more suitable model for carrying out polymer-like transformations. Subsequently, a series of derivatives was synthesized based on LMW chitosan by reductive alkylation (via the formation of Schiff base intermediates): these derivatives contain hydrophobic 4-tetradecyloxybenzyl and hydrophilic 4-(*N*,*N*,*N*-trimethylammonium)benzyl substituents on the polymer units. The introduction of hydrophobic functional groups confers amphiphilic properties on the new compounds, manifested in their ability to self-assemble into nanoscale structures. Hydrophobic chitosan derivatives are characterized by their ability to self-assemble into aggregates with diameters ranging from 80 to 360 nm, which are capable of effectively encapsulating and delivering lipophilic biologically active compounds [7]. By varying the type and degree of substitution of the hydrophobic moiety, it is possible to control the size and charge of the nanoobjects, as well as the release profile of the active substance [8,9].

Most chitosan samples near neutral pH have low solubility/dispersibility. The introduction of quaternary ammonium groups solves this problem—they carry a constant positive charge that is independent of external pH conditions, ensuring the stability of the nanoscale structures at the physiological pH of 7.4 and enabling the delivery of hydrophobic bioactive substances into the human body [10,11,12].

The increased positive surface charge of such nanoaggregates can be used to form complex polyelectrolyte complexes with proteins, nucleic acids, or polyanions, and to design multicomponent nanoscale carriers for bioactive substances [13,14]. Chitosan and its derivatives are being actively investigated as DNA and RNA delivery vectors for cancer therapy. Despite their lower efficacy compared to viral vectors, such polysaccharide vectors offer several advantages: safety, stability, and flexibility in chemical design, as well as the absence of undesirable inflammatory and immune reactions [15,16].

The aim of this study was to synthesize and characterize the structures of the resulting derivatives, and to evaluate the effect of the substituent ratio on the turbidity, charge, and ability of the compounds to self-assemble into nanoscale structures in aqueous media, including under physiological (pH 7.4) conditions.

## 2. Materials and Methods

### 2.1. Materials

The following details are relevant: chitosan molecular weight (MW) 1040 kDa, degree of deacetylation (DD) 85%, (BioprogressSchelkovo, Moscow, Russia); glacial acetic acid, 65% nitric acid, hydrochloric acid, sodium hydroxide, 25% aqueous ammonia, ethanol, dimethylformamide (Reakhim, Moscow, Russia); heavy water (D_2_O), trifluoroacetic acid (Chemical Line, Saint Petersburg, Russia); 4-hydroxybenzaldehyde, potassium carbonate, hexane (KhimMed, Moscow, Russia); 1-bromotetradecane, sodium cyanoborohydride, phosphate-buffered saline tablets (Sigma, St. Louis, MO, USA). Tetrazolium hydrochloride was used in the MTT test (Sigma-Aldrich, St. Louis, MO, USA) and Dulbecco’s modified Eagle’s medium (DMEM) containing 7% of fetal bovine serum, glutamine, and penicillin/streptomycin (PanEco, Moscow, Russia). Cells were trypsinized using a trypsin/EDTA solution (PanEco, Moscow, Russia). Other reagents and solvents used in the study were of analytical grade.

### 2.2. Characteristics of Low-Molecular-Weight Chitosan and Its Derivatives

The MW of chitosan was determined by high-performance liquid chromatography using a GPC PSS NOVEMA Max analytical 1000 A column (PSS, Mainz, Germany). In the 0.1 M NH_4_, acetate buffer was + 0.2 M NaCl at 30 °C, pH = 4.5, and an elution rate of 1.0 mL/min. The control and analysis of chromatograms was carried out using Multikhrom version 1.6 software (LLC Ampersand, Moscow, Russia). Pullulans (Mw = 342, 1260, 6600, 9900, 23,000, 48,800, 113,000, 200,000, 348,000, and 805,000 Da) (PSS, Mainz, Germany) were used as calibration standards.

The DD of chitosan was determined by ^1^H NMR. Spectra of chitosan and its derivatives were recorded on a Bruker Avance 600 spectrometer (Bruker, Billerica, MA, USA) operating at a frequency of 600 MHz using a standard one-pulse zg sequence at 25–60 °C. Spectra were acquired with 8 scans, 4 dummy scans, a relaxation delay of 2.0 s, a spectral width of 20.03 ppm (12.02 kHz), and 143,882 acquired points.

IR spectra were recorded on a Perkin-Elmer 1420 spectrometer (Perkin-Elmer, Shelton, CT, USA) covering the frequency range from 4000 to 400 cm^−1^. The studied samples were prepared in KBr (Sigma, St. Louis, MO, USA).

HRMS analysis was performed on an Impact II QqTOF high-resolution mass spectrometer (Bruker Daltonik, Bremen, Germany) equipped with MnESI ion source (Newomics, Berkeley, CA, USA) with the following conditions: direct sample infusion at 5 μL/min, ESI ion source in positive mode, HV capillary at 4.5 kV, spray gas—nitrogen at 0.7 bar, dry gas—nitrogen at 5 L/min 250 °C, scan range *m*/*z* 50–1500, 1 Hz scan rate, and automatic internal calibration with sodium trifluoroacetate solution. Spectra were processed with Compass Data Analysis 5.1 (Bruker Daltonik, Bremen, Germany).

### 2.3. Preparation of Low-Molecular-Weight Chitosan

High-molecular-weight crab chitosan (MW 1040 kDa, DD 85%) was subjected to acid hydrolysis [17]. A sample of chitosan (10 g) was placed in a 500 mL round-bottomed two-neck flask. Diluted nitric acid was then added: 180 mL of distilled water and 20 mL of 65% HNO_3_. The reaction mixture was stirred at 70 °C for 7 h, after which it was left for 12–14 h at room temperature. The next day, the reaction mixture was filtered through a Schott glass filter (Por 2). The resulting precipitate was placed in 100 mL of distilled water and heated in a water bath until completely dissolved. The chitosan solution was filtered again. The filtrate was then precipitated using a 25% aqueous ammonia solution at pH 9. The resulting precipitate was dialyzed against distilled water (MWCO 14 kDa), monitoring electrical conductivity and changing the water multiple times. The dialyzed product was frozen and freeze-dried. A total of 5.6 g of LMW chitosan was obtained. The yield was ~56%. LMW chitosan characteristics were as follows: Mw 47 kDa, polydispersity index (PDI) = 1.8, DD 96%.

### 2.4. Synthesis of p-Tetradecyloxybenzaldehyde

In total, 0.1 mol of 4-hydroxybenzaldehyde, 0.12 mol of potassium carbonate, and 300 mL of dimethylformamide (DMF) were placed in a 1 L three-necked flask. The reaction mixture was heated to 80 °C while stirring. A total of 0.12 mol of alkyl bromide was added to the heated mixture over the course of one hour through a dropping funnel. The mixture was left for 8 h while stirring at 80 °C. The next day, 300 mL of distilled water was added, and the mixture was extracted with petroleum ether (3 × 300 mL). The organic layer was washed twice with a 10% NaOH solution and then washed with distilled water until the solution was neutral. Next, the organic layer was dried over anhydrous MgSO_4_ and concentrated on a rotary evaporator, yielding a pale yellow oil that crystallized at room temperature as a white solid. The yield was 90%. ^1^H NMR (600 MHz, CDCl_3_): δ 9.88 (s, 1H, CHO), 7.83 (d, J = 8.8 Hz, 2H), 6.99 (d, J = 8.7 Hz, 2H), 4.04 (t, J = 6.6 Hz, 2H, CH_2_), 1.91–1.74 (m, 2H, CH_2_), 1.50–1.43 (m, 2H, CH_2_), 1.26 (m, 20H, CH_2_), 0.87 (t, J = 6.8, 3H, CH_3_). HRMS (MnESI) *m*/*z* calculated for C_21_H_34_O_2_ + H^+^ 319.2637; found 319.2632.

### 2.5. Synthesis of N-[4-Tetradecyloxybenzyl]chitosan (H2.5–H10)

A sample of chitosan (4 g) was dissolved in 220 mL of a 0.2 M acetic acid (AcOH) solution. Once complete dissolution was achieved, 150 mL of ethanol (EtOH) was added. A solution of p-tetradecyloxybenzaldehyde in EtOH was added in the appropriate ratio (2.5–10 mol% to NH_2_ groups of chitosan) before adding an excess of sodium cyanoborohydride (NaCNBH_3_) (3 moles per 1 mole of aldehyde). The mixture was stirred for 24 h at RT, and the alkylated chitosan was precipitated with a 25% aqueous solution of ammonium hydroxide (NH_4_OH) under stirring. The pH of the solution was adjusted to 10. The precipitate was separated by centrifugation on a Sigma 4–16K centrifuge (SigmaLaborzentrifugen GmbH, Osterode am Harz, Germany) at 10 °C, 6000 rpm, 15 min) and washed 3–4 times with ethanol. The resulting precipitate was dialyzed (MWCO 14 kDa) against distilled water. The dialyzed product was frozen and freeze-dried. The yield was ~85–90%. The degree of substitution was determined using the following formula,

(1) $${DS}_{1}=\frac{\frac{1}{27}\times I(H10)}{\frac{1}{6}\times (I\left(H2-H6\right)}$$

as the ratio of the area under the signal of the aliphatic fatty tail H10 and the signals of H2–H6 of the pyranose ring of the chitosan units.

### 2.6. Preparation of the Quaternary Ammonium Salt—4-(N,N,N-Trimethylammonium)benzaldehyde Iodide

Iodomethane (CH_3_I) (42.3 g, 0.3 mol) was added to a solution of 4-(dimethylamino)benzaldehyde (14.9 g, 0.1 mol) in acetone (50 mL). The solution was stirred for 8 h at 70 °C with a reflux condenser. During the reaction, a salt—4-formyl-*N,N,N*-trimethylbenzenaminium iodide precipitated from the solution. The precipitate was separated by filtration, washed with diethyl ether, and the residual solvent was removed on a rotary evaporator. The yield was 80%. ^1^H NMR (600 MHz, DMSO-d6): δ 3.64 (s, 9H, CH_3_), 8.15 (d, 2H, Ph-H, J = 8.7 Hz), 8.21 (d, 2H, Ph-H, J = 8.7 Hz), 10.10 (s, 1H, CHO); HRMS (MnESI) *m*/*z* calculated for C_10_H_14_NO+ 164.1075, found 164.1070.

### 2.7. Synthesis of N-[4-(N,N,N-Trimethylammonium)benzyl], [4-Tetradecyloxybenzyl]chitosan Chloride

A weighed portion (500 mg) of chitosan derivative (H2.5–H10) was dissolved in 50 mL of 0.2 M AcOH. A sample of 4-(*N,N,N*-trimethylammonium)benzaldehyde iodide (6–25 mol% to NH2 groups of derivatives) was dissolved in 5 mL of water and added dropwise to the solution of the hydrophobic chitosan derivative while stirring vigorously with a magnetic stirrer. The reaction mixture was stirred at RT for 4 h. Then, a threefold excess (relative to the aldehyde) of an aqueous solution of NaCNBH_3_ was added in portions. The mixture was stirred for 24 h at RT. The resulting polymer was purified by dialysis against a 1% NaCl solution for two days, followed by dialysis (MWCO 14 kDa) against distilled water for another three days, and then freeze-dried [18]. The yield was ~92–95%. Since low degrees of quaternization result in a poor signal-to-noise ratio for the integration of anomeric or aromatic signals, the degree of substitution (DS_2_) was therefore determined based on the most intense signal—the trimethyl signal at 3.78 ppm. The trimethylammonium proton signal I(Q) overlaps with the H2-H6 region of the chitosan. Therefore, the total integral ∑I of this region contains 6 protons per monosaccharide unit and an additional 9 protons contributed by the trimethylammonium groups. Accordingly, the degree of substitution can be expressed as ${DS}_{2}=\frac{I\left(Q\right)/9}{\sum I/(6+9\times {DS}_{2})}$. So it was calculated using the following formula:

(2) $${DS}_{2}=\frac{6\times I(Q)}{9\times (\sum I-\;I\left(Q\right))}$$

where I(Q) is the integral of the trimethyl protons, and ∑I is the total integral of the overlapped glucopyranose and trimethyl proton regions.

### 2.8. Turbidimetric Titration of Derivative Solutions

The dependence of the sample’s turbidity in water on pH was evaluated by measuring turbidity, similar to the method used by Caiqin Qin, Qi Xiao, et al. in their study [19]. A total of 20 mg of the tested sample was dissolved in 10 mL of a 1% AcOH solution. While gradually adding a 0.1 M NaOH solution, the absorbance of the derivative solutions was measured at pH values ranging from 3 to 12 using a Leki SS1207 spectrophotometer (LEKI Instruments, Helsinki, Finland) at 600 nm.

### 2.9. Determination of Particle Size and ζ-Potential

Sample solutions for particle size (hydrodynamic radius) determination were prepared in 0.5% AcOH and 0.01 M PBS, 0.0027 M KCl, 0.137 M NaCl at concentrations of 5 mg/mL. The charge was measured in 0.5% AcOH and a diluted (0.001 M PBS) solution at a concentration of 2.5 mg/mL. Measurements were performed on a Photocor Compact-Z analyzer (Photocor, Moscow, Russia) using the Photocor and Photocor Zeta software (version 7.37.19.145), with a glass cylindrical cuvette. All measurements were performed using a 638 nm laser light at 25 °C with a 90° angle of detection.

### 2.10. Cytotoxicity Analysis of Chitosan Derivatives

To assess the cytotoxicity of chitosan derivatives, MTT analysis was performed on SH-SY5Y, BT474, Skov3 cell lines [20]. Cells were cultured in DMEM medium and trypsinized using trypsin/EDTA solution. The solutions were diluted to reduce their concentration from 500 μg/mL to 2 μg/mL. Cells were seeded at a concentration of 10,000 cells/well, then 100 µL of DMEM (containing 7% FBS) was added and incubated at 37 °C in the presence of 5% CO_2_ for 72 h. MTT reagent was added to each well for the last 4 h. The culture medium was removed from the wells, and formazan crystals were dissolved in 100 μL DMSO for 20 min. Optical density was measured at 540 nm on a Multiskan FC plate spectrophotometer (Thermo Fisher Scientific, Waltham, MA, USA), and the growth inhibition index was calculated (3) as follows:

(3) $$Inhibition\;index=1-\frac{Sample\;{OD}_{540}}{Control\;{OD}_{540}}$$

where Sample OD_540_ and Control OD_540_ are the optical densities of the sample and control, respectively. The experiments were carried out in triplicate.

## 3. Results and Discussion

**Figure 1:**
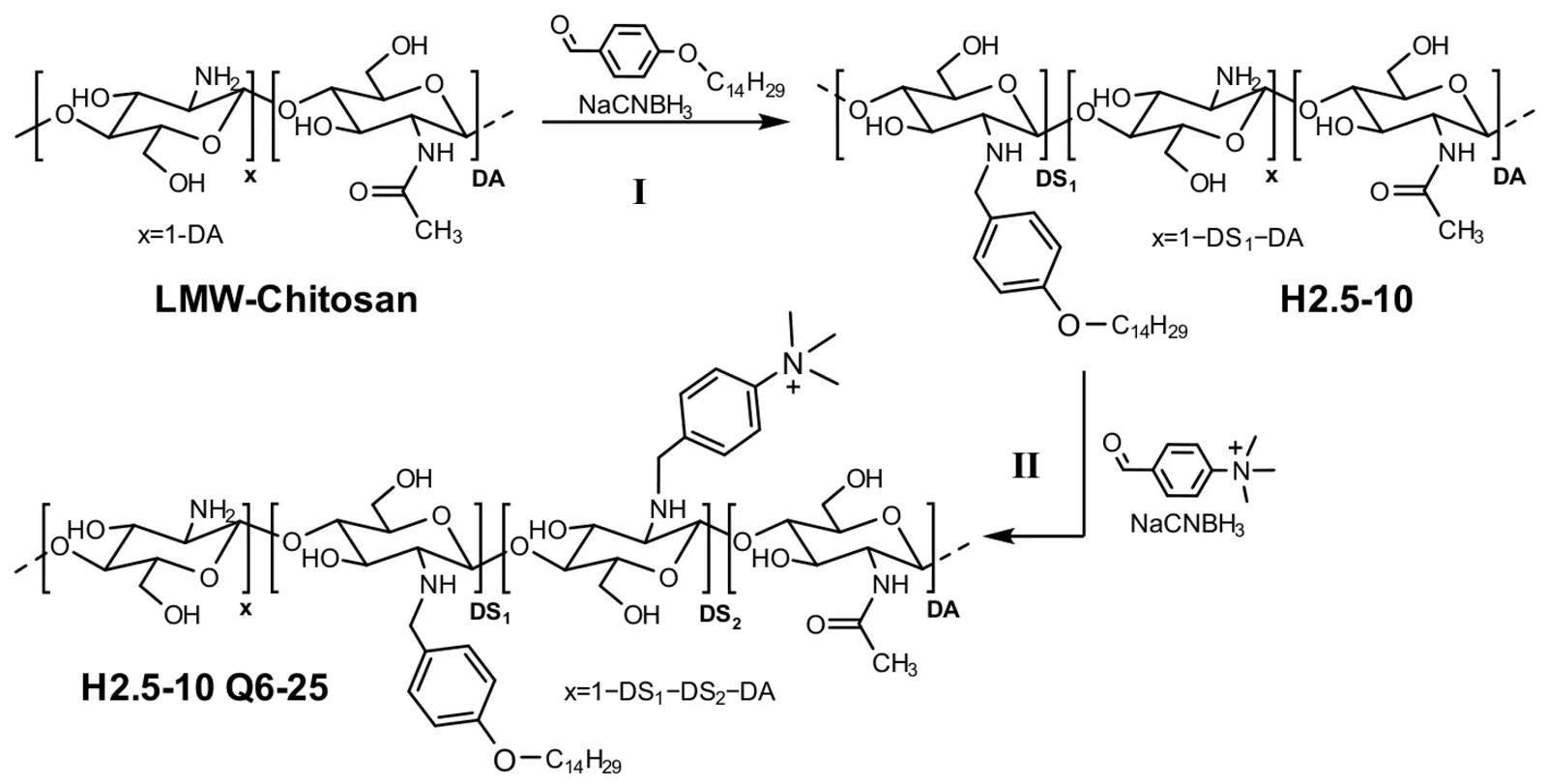
Overall scheme for the synthesis of amphiphilic chitosan derivatives.

Chitosan derivatives were synthesized in two stages: I—reductive alkylation of chitosan with 4-tetradecyloxybenzaldehyde; II—reductive alkylation of H2.5–10 derivatives with 4-(*N*,*N*,*N*-trimethylammonium)benzaldehyde. The general synthesis scheme is shown below in Figure 1.

### 3.1. Alkylation of Chitosan with a Hydrophobic Substituent

**Figure 2:**
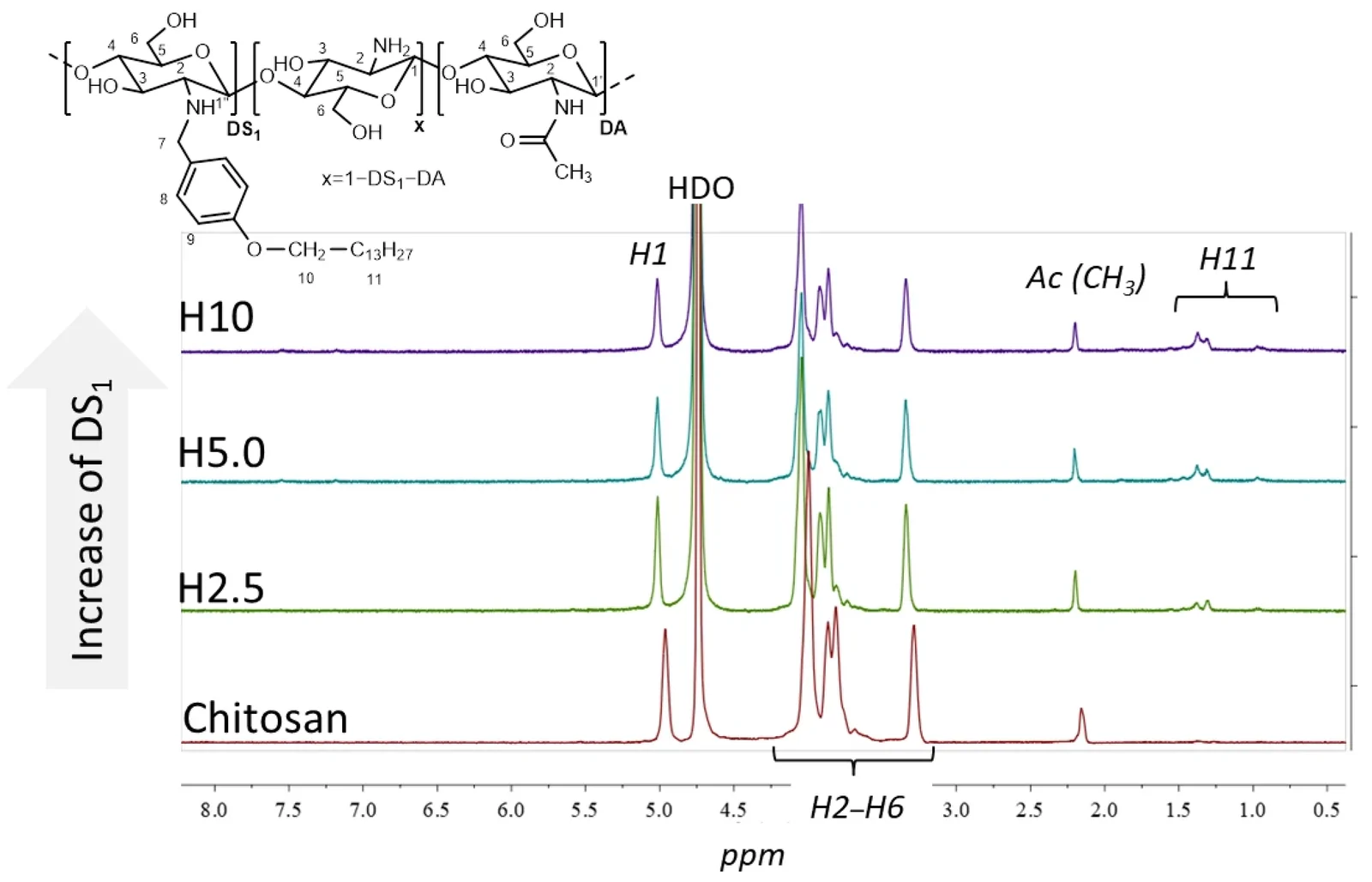
Stack of ^1^H-NMR spectra of hydrophobic derivatives of low-molecular-weight chitosan (CF_3_COOH/D_2_O—1/20, 60 °C).

As a result of the alkylation of chitosan with C14-aldehyde, followed by a reduction in the imine with NaCNBH_3_, new signals appeared in the high-field region from the C14 protons of the –(CH_2_)_n_– chain and a weak signal from the CH_3_ terminal fragment at 0.87–0.9 ppm [21] from the oxybenzyl substituent. As the degree of substitution increased, signals from the symmetric aromatic system also became noticeable at approximately 7.5–7.0 ppm. Stack of ^1^H NMR spectra of the derivatives are shown in Figure 2.

Based on NMR data, the following degrees of substitution were obtained: 0.5, 1.3, and 4.1%. The results are presented in Table 1.

### 3.2. Introduction of a Cationic Substituent into Hydrophobically Modified Chitosan

An approach combining the introduction of hydrophobic and cationic substituents—hydroxypoltrimethylammonium and a palmitic (C16) acid residue—was previously used in [22]. The derivative was obtained by “grafting” palmitic acid, activated with water-soluble carbodiimide, onto quaternized chitosan. The heterogeneity and non-selectivity of the methods used *(N, O)* mean that the relationship between the compound’s structure and the amphiphilic properties of the macromolecules remains unclear [23].

**Figure 3:**
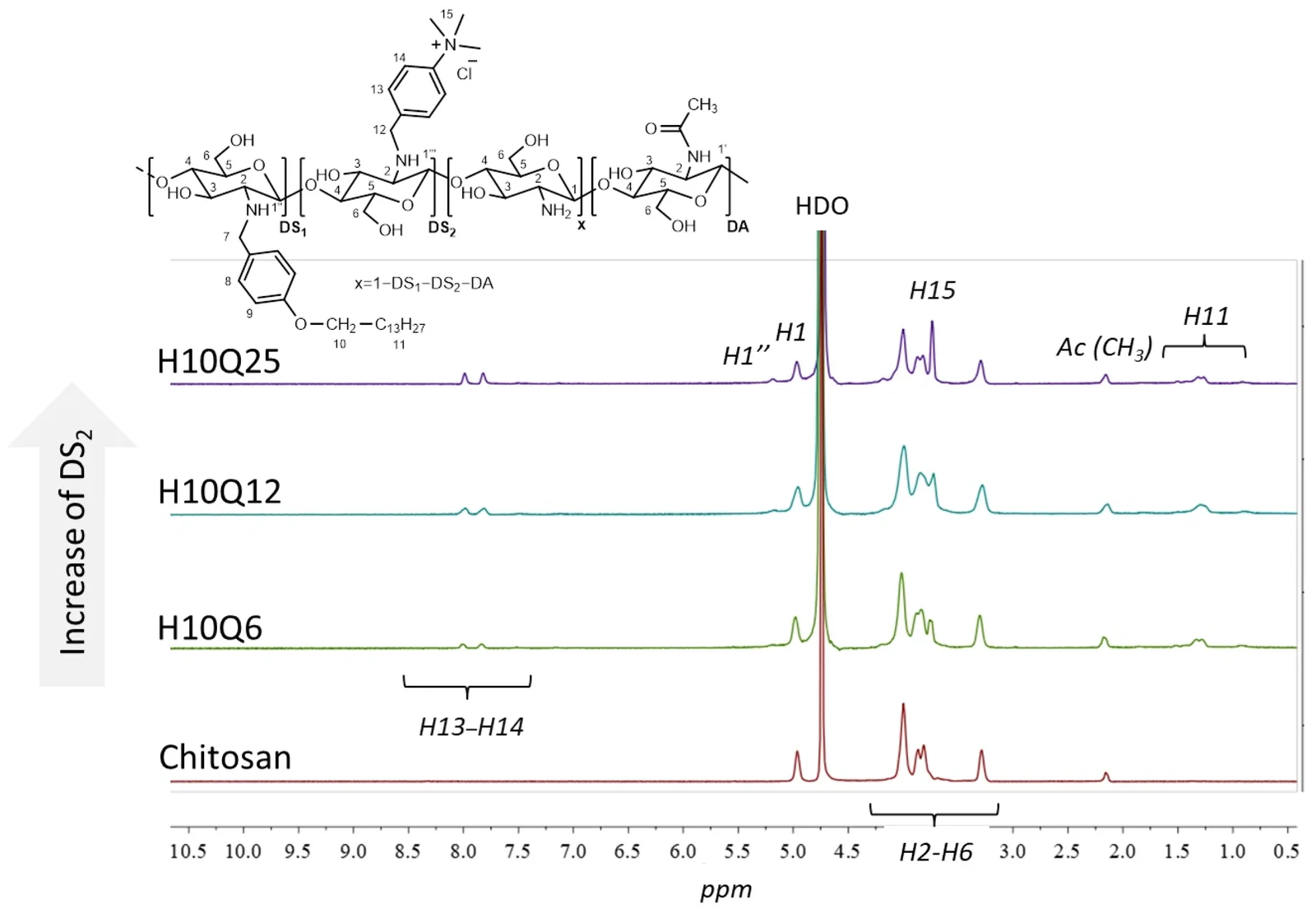
Stack of ^1^H-NMR spectra of hydrophobic derivatives with an additional quaternized moiety (CF_3_COOH/D_2_O—1/20, 60 °C).

The hydrophobic derivatives H2.5–10 obtained at the first stage were further modified with a quaternary ammonium group, also through reductive alkylation. The final compound has a defined structure—it is the product of selective *N*-substitution of low-molecular-weight chitosan, which makes the nanoscale system a much more straightforward platform for evaluating the effect of substituent composition on the system’s properties. As a result of the alkylation of chitosan with Q-aldehyde, new signals appeared in the low-field region from the symmetric protons of the benzyl substituent and the trimethyl protons of the quaternary ammonium salt *(H15)* (Figure 3). According to NMR data, the degrees of substitution of the derivatives ranged from 6 to 18%. The results are presented in Table 2. The lower amino-group conversion observed at 25% mol eq. of aldehyde may be attributed to the reduced accessibility of free amino groups caused by hydrophobic self-association of the polymer chains and electrostatic repulsion between the cationic aldehyde and the positively charged surface of the aggregates.

### 3.3. IR Spectroscopy

**Figure 4:**
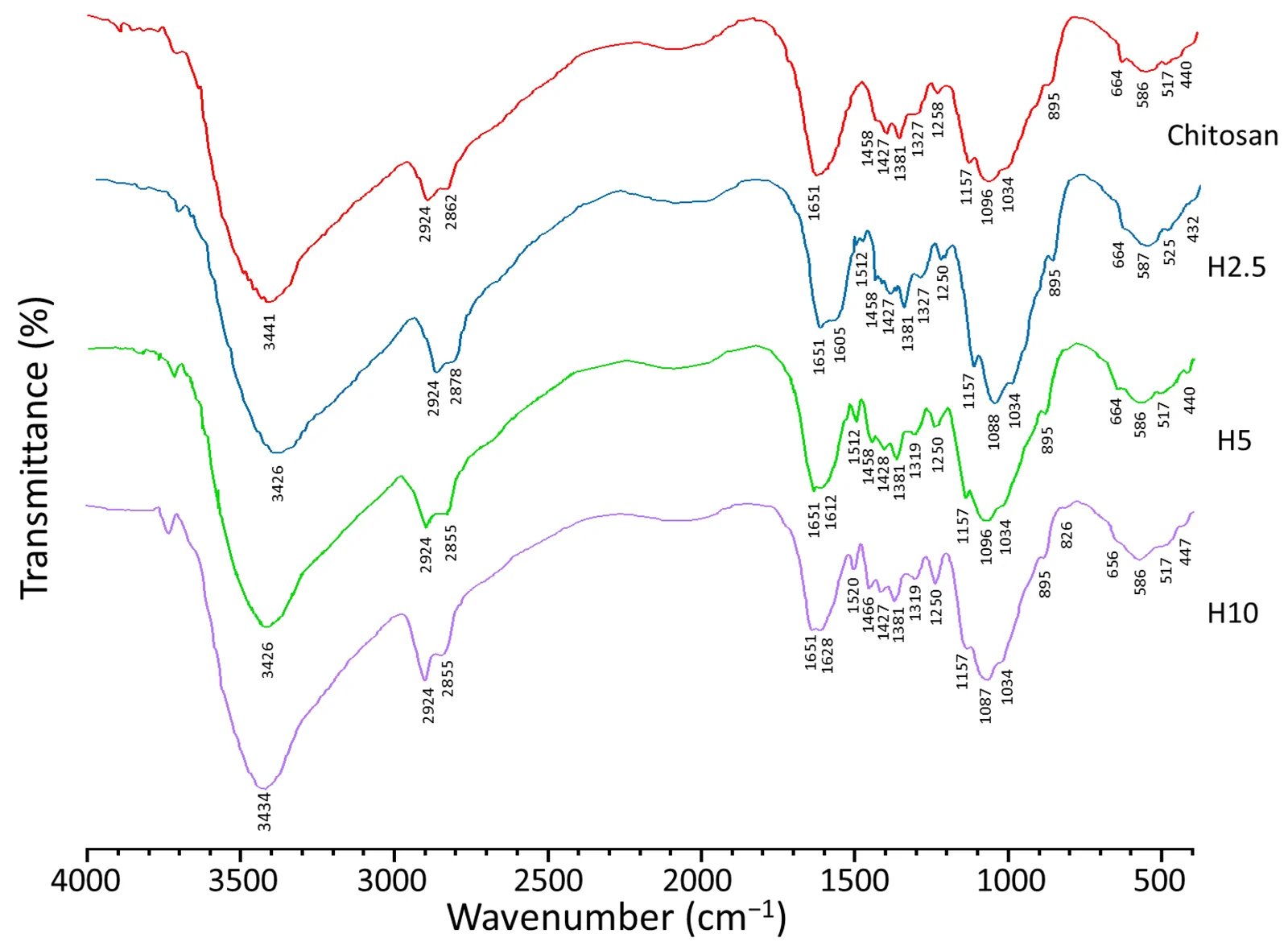
Stack of IR spectra of chitosan and its hydrophobic derivatives H2.5–H10.

**Figure 5:**
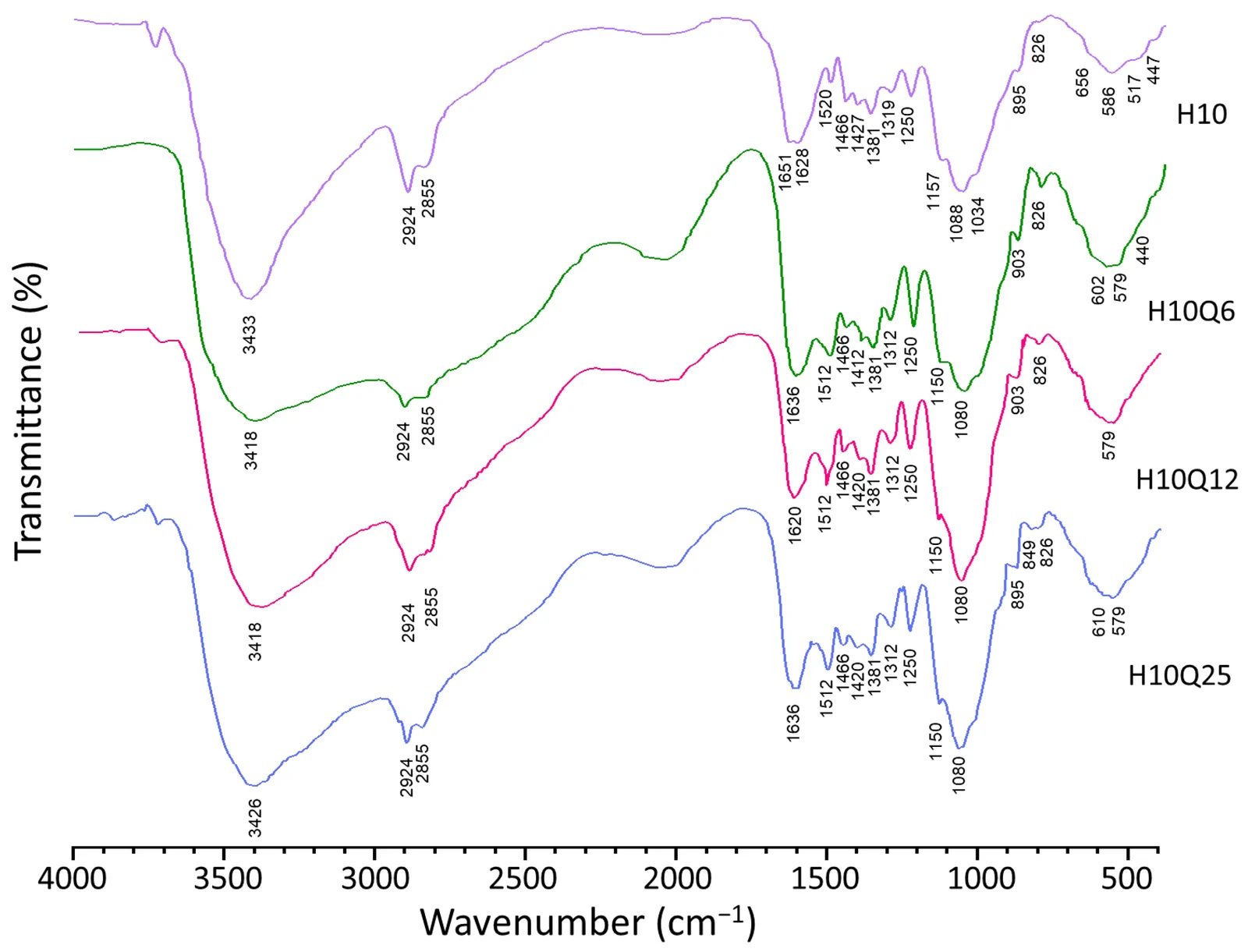
Stack of IR spectra for the hydrophobic derivative H10 and the disubstituted derivatives H10Q6-25.

The results of IR spectroscopic analyses of chitosan and its derivatives are presented in Figure 4 and Figure 5. The IR spectrum of low-molecular-weight chitosan shows characteristic chitosan bands: the OH/NH stretching vibrations at 3450 cm^−1^, the C–H valence vibration bands in the range of 2800–2900 cm^−1^, the δ vibrations N–H in the amino groups at 1620–1630 cm^−1^, -CH_2_- bending vibrations centered at 1420 cm^−1^; valence vibrations at 1315–1320 cm^−1^ (amide III from residual acetyl groups), skeletal vibrations (γ) C-O-C, including a valence vibration band at 1030 or 1070 cm^−1^ and antisymmetric vibrations (st as) near 1160 cm^−1^ [24].

When chitosan is modified with a hydrophobic substituent, the following changes are observed in the IR spectrum: the peaks in the 2800–2900 cm^−1^ range (C–H st) become sharper, and the peak at 1250 cm^−1^ also becomes sharper as the hydrophobic moiety (phenyl-alkyl C–O st) increases (Figure 4). Upon further quaternization of the hydrophobic chitosan (Figure 5), the following changes are observed in the IR spectrum: the band centered at 3450 cm^−1^ (N–H/O–H st) broadens, and the band change in the 1500–1640 cm^−1^ region (N–H δ) may result due to *N*-substitution of chitosan with quaternary fragment. A new peak appears at 1466 cm^−1^, which is attributed to the presence of a quaternary ammonium group [19]. In both cases, spectral shifts are observed due to the presence of identical structural fragments (R-Bn-) in the introduced groups. For example, both substituents result in *N*-substitution and therefore change the 1620–1630 cm^−1^ band region (–NH– δ from –NH_2_ δ). Additional changes in the broad 1500–1650 cm^−1^ region may arise from overlapping contributions of amino-group deformation, residual amide groups, bound water, and aromatic ring vibrations. Compared to chitosan, the peaks corresponding to the vibrations of the skeletal polysaccharide oxygen bridges, including C–O stretching, occurring between 1154 and 1079 cm^−1^, remained unchanged in the chitosan derivatives. The above-mentioned changes indicated the attachment of new fragments to the –NH_2_ group of the chitosan unit after reductive alkylation [25].

### 3.4. Turbidimetric Titration of Derivative Solutions

**Figure 6:**
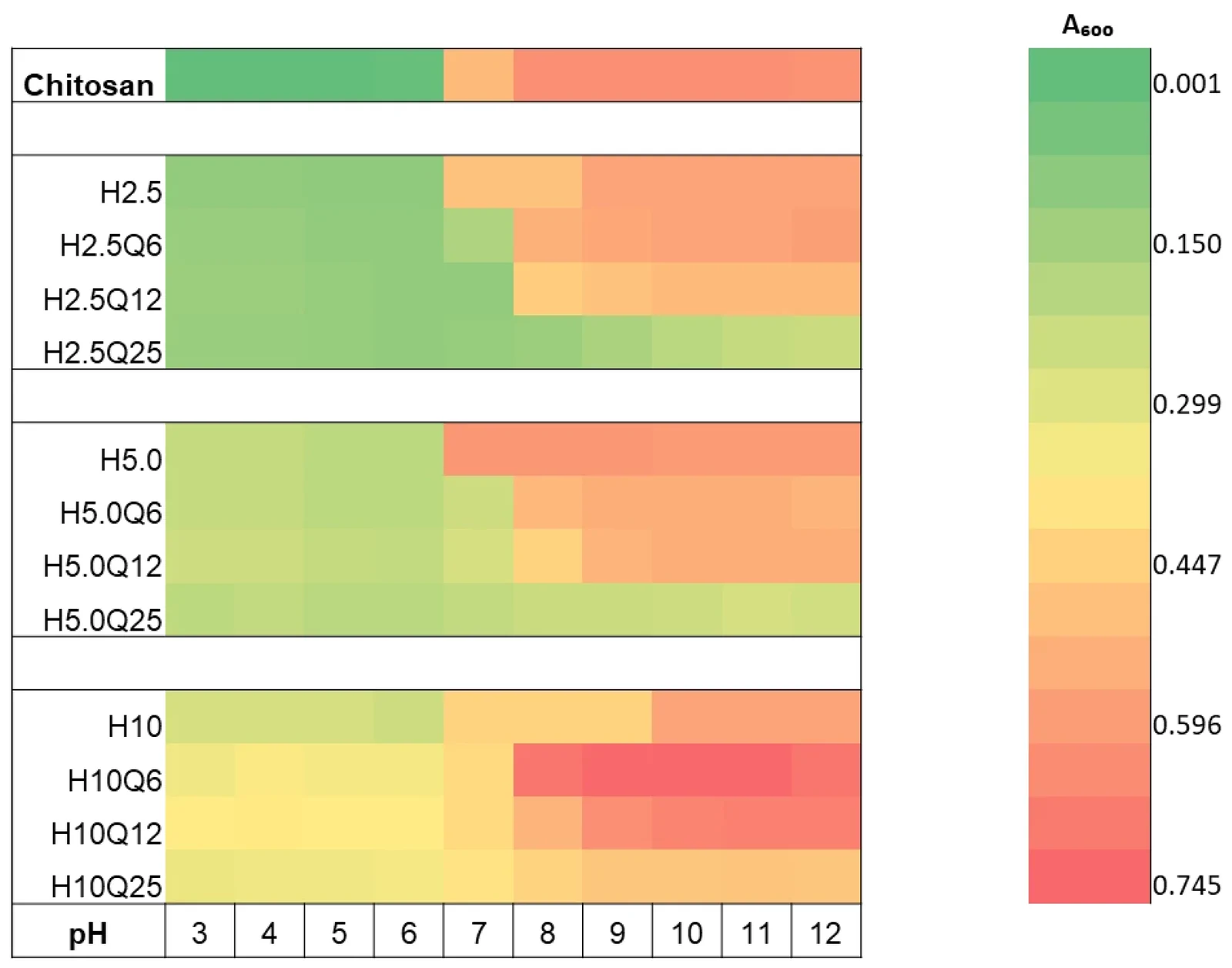
Heatmap of turbidimetric titration of solutions/dispersions of chitosan derivatives at a concentration of 2 mg/mL. Abs_600_ corresponds to absorbance at 600 nm.

Turbidimetric titration of the solutions was performed to evaluate the pH-dependent behavior of the derivatives in an aqueous medium (Figure 6). Chitosan is soluble in water only under acidic conditions (pH < 7)—an increase in solution turbidity was observed upon transition to the alkaline range. For derivatives H2.5, H5.0, and H10, an increase in Abs_600_ was observed in the pH range of 3–6, associated with the aggregation of new C14-hydrophobic substituents in the derivatives. Upon further transition into the alkaline range (pH > 7), as DS_1_ increases, the turbidity of the solutions decreases slightly compared to that of chitosan in the control. These data correlate with the results for acylated chitosans obtained by Chirano and co-authors—the solubility of hydrophobic derivatives may improve under alkaline conditions [26].

In the classic single-substituent derivatives, chitosan exhibits increased solubility after quaternization due to the presence of quaternary ammonium groups, whose charge is independent of pH [27]. A similar pattern is characteristic of a number of H2.5 derivatives. An increase in the degree of quaternization (DS_2_) reduces their aggregation in aqueous solution. This is most clearly observed for the most substituted derivative in the H2.5Q25 series. For the series of derivatives with moderate hydrophobicity (H5.0), quaternization similarly improved apparent turbidity as DS_2_ increased.

An interesting pattern was observed for the most hydrophobic H10 derivatives. A solution of the derivative in an acidic medium is characterized by aggregation, which is enhanced by the addition of a small amount of the quaternized DS_2_ fragment—6% and 12% for the H10Q6 and H10Q12 derivatives, respectively—especially in an alkaline medium. This effect was not observed for the chitosan derivative with the highest DS_2_ value—H10Q25.

In summary, it can be said that in most cases, an increase in quaternization reduces the aggregation of derivatives in solution; however, the effect of substituents exhibits a nonlinear relationship and is determined by the balance between the hydrophilicity and hydrophobicity of the derivatives, which is regulated by the combined introduction of different types of substituents [26,28]. Thus, a high degree of quaternization and moderate content of hydrophobic substituents in H5.0Q25 ensure consistently low turbidity of the chitosan derivative across the entire pH range of 3–12. However, in the case of H10Q6, the cationic substituent contributes to the strongest aggregation of the derivatives in the alkaline region among all synthesized samples (13 samples including chitosan). Korchagina and Philippova [29] showed that low-molecular-weight chitosan can form multichain aggregates with a comparatively high aggregation number, where electrostatic repulsion plays an important role in limiting aggregate growth. In our case, the lowest permanent cationic substitution in this series may be insufficient to maintain strong electrostatic repulsion to prevent hydrophobic association after deprotonation of residual amino groups. The resulting unusually high turbidity may reflect the incorporation of a larger number of polymer chains in individual aggregates of H10Q6 while still providing a permanent charge to prevent precipitation. This balance may favor pronounced rearrangement of the amphiphilic polymer into larger and relatively stable aggregates than compared with H10, which lacks permanent charge.

### 3.5. Particle Size and Charge of Chitosan Derivatives

**Figure 7:**
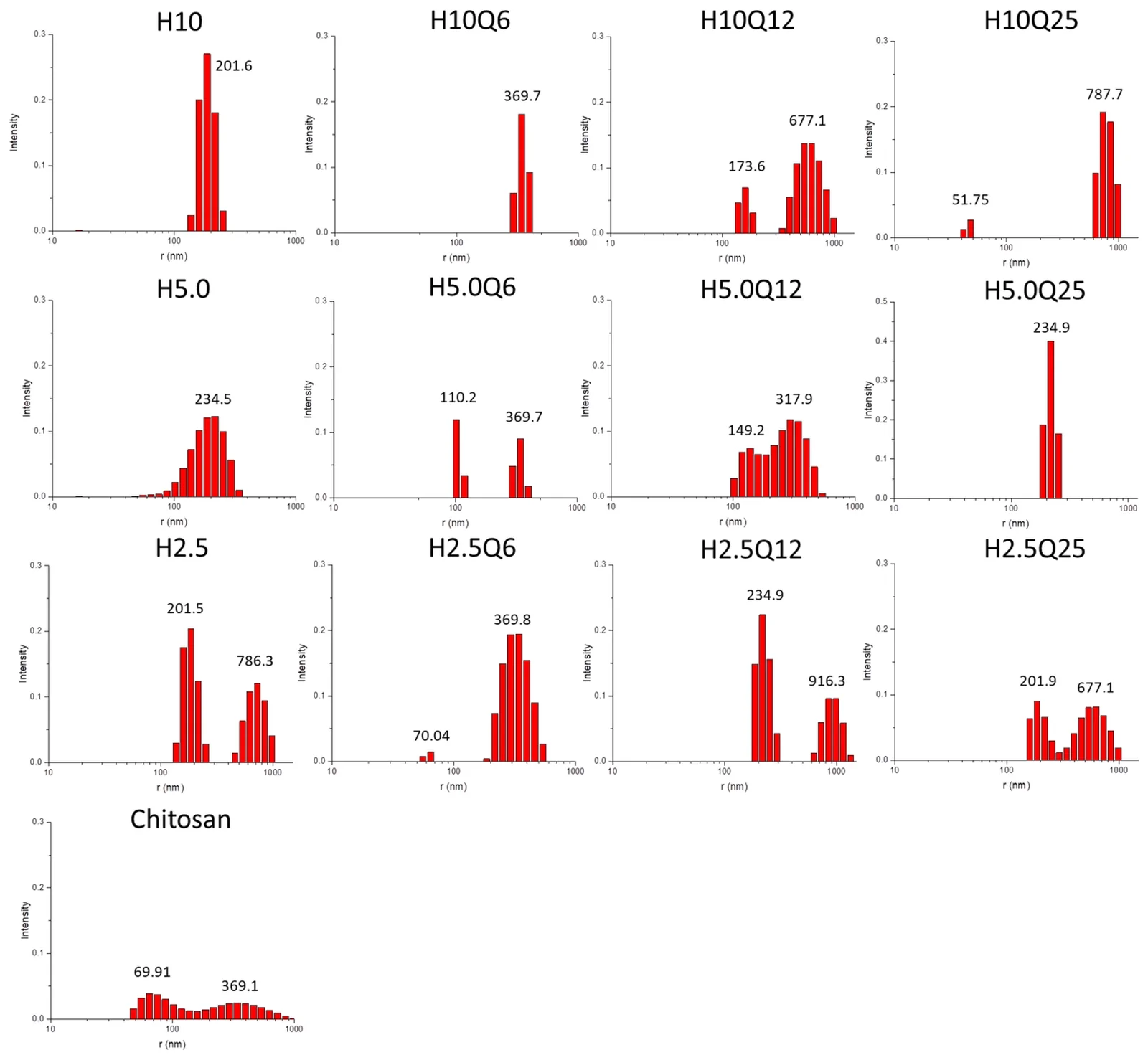
Particle size distributions (by intensity) obtained using the DLS method (scattering angle 90°, t 25 °C) for samples (5 mg/mL) in 0.5% AcOH.

**Figure 8:**
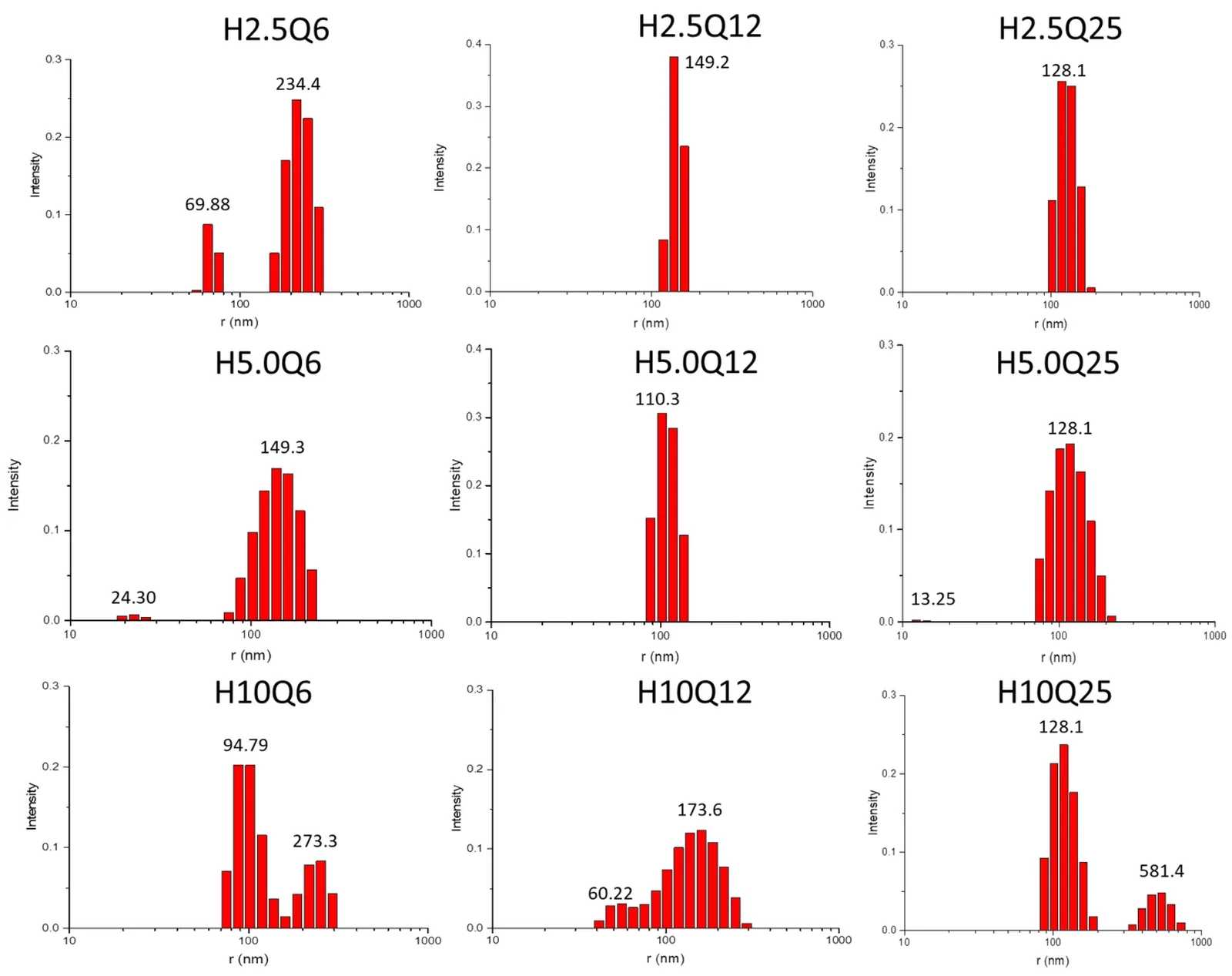
Particle size distributions (by intensity) obtained using the DLS method (scattering angle 90°, t 25 °C) for samples in 0.01 M PBS.

The particle size of the derivatives was studied in 0.5% AcOH (Figure 7, Table 3) and under neutral conditions—in a phosphate–saline buffer consisting of 0.01 M PBS, 0.0027 M KCl, 0.137 M NaCl at pH 7.4 (Figure 8, Table 4). A comparison of the characteristics of the aggregates under different conditions allows us to trace changes in the nature of the aggregates, which are stabilized by the positive charge of the amino group in an acidic medium and by quaternary ammonium substituents under neutral conditions [30,31,32].

#### 3.5.1. Study Under Acidic Conditions

In an acidic medium, the initial low-molecular-weight chitosan formed two main aggregate fractions with broad distributions, exhibiting two main peaks at 70 and 369 nm. The LMW chitosan aggregates had a positive ζ-potential of +16.8.

Introduction of a hydrophobic substituent. As a result of introducing a C14 hydrophobic substituent into the chitosan structure, a characteristic peak appeared at 200–230 nm, and the ζ-potential increased to +27–29 mV for the H2.5–10 derivatives. For the H2.5 derivative with a substitution degree of 0.5%, the formation of an additional large particle fraction with an average radius of 786 nm was observed, indicating the formation of large macromolecular associates at a low substitution degree (DS_1_) [33].

Introduction of a cationic substituent. The introduction of a cationic substituent increased the ζ-potential of the nanoaggregates to +44.6 mV. The highest value was observed for the H10Q25 derivative (DS_1_ = 4.1%, DS_2_ = 18%), where, on the one hand, the association of hydrophobic tails and, on the other hand, the increased density of cationic groups increases the charge of the particles. In general, for hydrophobic derivatives, the effect of the additional cationic substituent was not monotonic. For H2.5, a significant increase in the proportion of larger particle intensity was observed for H2.5Q6 and H2.5Q25. Achieving the highest degree of quaternization for H5.0 led to a unimodal particle distribution with a hydrodynamic radius of 235 nm. Similar behavior was observed for the H10Q6 sample but with a larger radius of 370 nm.

#### 3.5.2. Study Under Neutral Conditions

Under PBS conditions, a clear decrease in the charge of the nanoaggregates was observed (Figure 8, Table 4). All amphiphilic chitosan derivatives in the HQ series exhibited a positive ζ-potential (+17–25 mV), confirming that the cationic nature of the aggregates was preserved under physiological conditions at pH 7.4 due to the introduction of new cationic substituents [12]. For some derivatives, there has been a transition from a bimodal to apparently unimodal distribution, for example, for the H2.5Q6-25 series. For the H5.0Q12 derivatives, the most compact aggregates were observed, with a hydrodynamic radius of 110 nm. For the H10 derivatives, which were most substituted on the hydrophobic C14-tail, the size distributions retained a bimodal character. The presence of the largest particle fraction in H10Q25 indicates that the introduction of 18% cationic substituent cannot fully compensate for the hydrophobic association of 4% C14 groups. An increase in DS_2_ clearly leads to a decrease in particle size only in the case of 0.5% C14 groups. In all other cases, a redistribution of scattering intensity occurs between compact and large aggregates. In addition, it is worth noting that when measuring particle size under neutral conditions, a decrease in the micron-sized fraction intensity is observed, in contrast to acidic conditions.

Particle size and ζ-potential are the main characteristic parameters of nanosuspensions. The ζ-potential is a measure of surface charge and can significantly affect the stability of particles in solution due to interparticle electrostatic repulsive forces. Suspensions are considered stable at a ζ-potential greater than +15 mV. However, ζ-potential alone is insufficient to predict long-term dispersion stability [34,35].

### 3.6. Cytotoxicity of Chitosan Derivatives

**Figure 9:**
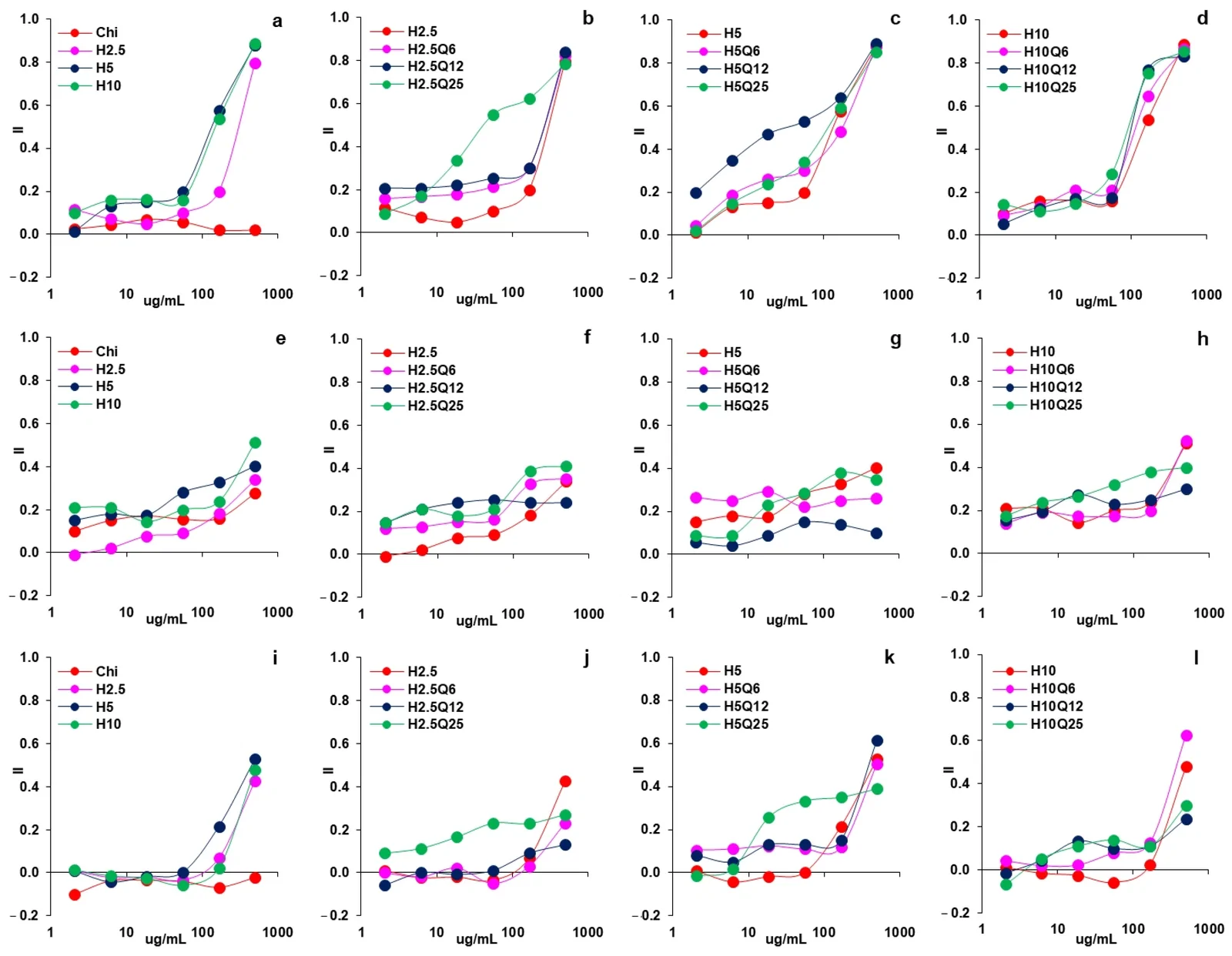
Cytotoxicity of the synthesized chitosan derivatives on cell lines: (**a**–**d**)—SH-SY5Y, (**e**–**h**)—BT474, (**i**–**l**)—Skov3. II—growth inhibition index.

The primary cell-response screening of chitosan derivatives was studied using the following cell lines: SH-SY5Y neuroblastoma, BT474 breast cancer, and Skov3 ovarian cancer. The results presented demonstrate that the synthesized amphiphilic quaternized chitosan derivatives exhibit differentiated cytotoxic activity (Figure 9).

The most pronounced cytotoxic effect is observed for samples with high degrees of substitution by both hydrophobic (C14) and cationic (Q) groups. In particular, sample H10Q25 (DS_1_ = 4.1%, DS_2_ = 18%) exhibits high cytotoxicity across all three cell lines. In contrast, sample H2.5Q6 (DS_1_ = 0.5%, DS_2_ = 6%) exhibits, on average, the lowest cytotoxic activity, indicating the dose-dependent nature of the effect.

The SH-SY5Y neuroblastoma cell line (Figure 9a–d) exhibits high sensitivity to chitosan derivatives at high concentrations of 167–500 μg/mL. The BT474 breast cancer cell line (Figure 9e–h) exhibits moderate sensitivity (no more than 40% inhibition at a sample concentration of 500 μg/mL). For the Skov3 ovarian cancer cell line (Figure 9i–l), a high degree of variability in response was noted: at low concentrations, almost all samples, except H10Q25, slightly increase cell proliferation; however, as the concentration and DS increase, they exhibit a pronounced cytotoxic effect, which correlates with data on the sensitivity of Skov3 cells to chitosan [36]. The slight increase in Skov3 cell proliferation observed at low concentrations of the derivatives may reflect a nonlinear, biphasic cellular response. Similar hormetic dose–response patterns have been reported for various nanomaterials [37], whereby low-level exposure stimulates cellular responses, whereas higher exposure levels lead to inhibition or cytotoxic effects. However, the present results should be interpreted with caution, as the underlying mechanisms were not investigated; therefore, it cannot be concluded that the observed response is hormetic. The concentration-dependent cytotoxicity observed for the more highly substituted derivatives is consistent with the assumption that cellular responses are influenced by the balance between hydrophobic interactions and cationic surface interactions.

The results obtained allow us to establish a relationship between the DS and cytotoxic activity. An increase in the C14-tail content from 0.5% to 4.1% leads to increased cytotoxicity in all the cell lines studied. This can be explained by the enhanced amphiphilic properties of the derivatives, which promote their self-assembly into nanoparticles and more effective interaction with cell membranes [38]. Similarly, an increase in the degree of quaternization from 6% to 18% correlates with an increase in the cytotoxic effect. Positively charged quaternary ammonium groups interact electrostatically with negatively charged cell membranes, which enhances the adsorption of the polymer onto cell surfaces and the subsequent disruption of membrane integrity [39,40]. The observed dose-dependent cytotoxicity is consistent with studies showing that quaternized chitosan derivatives exhibit antiproliferative activity against various cancer cell lines. In a study by Phuangkaew et al., it was shown that amphiphilic quaternized chitosan derivatives exhibit better water solubility and biological activity compared to unmodified chitosan [38].

However, the relationship between the DS and cytotoxic activity was not uniformly monotonic across all tested conditions. These observations indicate that increasing the degree of hydrophobic or cationic substitution does not necessarily enhance cytotoxicity under all conditions. The biological response may depend on the combined substitution pattern and its effects on polymer self-association, aggregate characteristics, and interactions with cell membranes, as well as on the sensitivity of the individual cell line.

## 4. Conclusions

The simultaneous introduction of hydrophobic C14 fragments and cationic substituents allows for the regulation of pH-dependent aggregation of low-molecular-weight chitosan derivatives. The introduction of both substituents was confirmed by ^1^H-NMR spectroscopy. The actual degree of substitution was 0.5–4.1% and 6–18% for the hydrophobic and cationic fragments, respectively. The hydrophobic fragments in the structure of the derivatives promote nanoscale association of macromolecules, while the cationic fragments stabilize the resulting aggregates. Turbidimetric titration demonstrated that the properties of the system do not change linearly with an increase in the content of a single substituent but depend on the balance between the two different types of modifications. All amphiphilic derivatives retained a positive charge under physiological conditions (PBS, pH 7.4). The degrees of hydrophobic and cationic substitution influence the cytotoxic activity of the derivatives; however, this effect is not uniformly monotonic and depends on the substitution pattern and the cell line. The results of the study show that varying the content of each substituent in the structure of the derivatives allows for the regulation of pH-dependent macromolecular aggregation in aqueous solution while maintaining the positive charge of the nanoaggregates. Thus, the chitosan derivatives studied represent a promising platform for a potential nanocarrier/drug delivery platform with the ability to modulate biological activity by altering the degrees of substitution. An important next step is to evaluate these derivatives as carriers of hydrophobic compounds, using a model drug such as curcumin to assess encapsulation efficiency, loading capacity, stability, and release behavior. However, further studies are needed to establish the drug-loading performance.

## Figures and Tables

**Table 1:** Characteristics of hydrophobic low-molecular-weight chitosan derivatives.

№	Code	C14-Aldehyde in the Reaction Mixture, Molar % of NH_2_ Groups	DS_1_ (%) by NMR
1	Chitosan	0	0
2	H2.5	2.5	0.5
3	H5.0	5.0	1.3
4	H10	10	4.1

**Table 2:** Composition and degrees substitution of amphiphilic quaternized/hydrophobic derivatives of low-molecular-weight chitosan.

№	Code	C14-Aldehyde in the Reaction Mixture, Molar % of NH_2_ Groups	DS_1_ (%) by NMR	Q-Aldehyde in the Reaction Mixture, Molar % of NH_2_ Groups	DS_2_ (%) by NMR
1	H2.5Q6	2.5	0.5	6	6
2	H2.5Q12			12	11
3	H2.5Q25			25	17
4	H5.0Q6	5.0	1.3	6	6
5	H5.0Q12			12	12
6	H5.0Q25			25	17
7	H10Q6	10	4.1	6	6
8	H10Q12			12	12
9	H10Q25			25	18

Here and throughout, in the sample codes, the first number corresponds to the nominal amount of C14-aldehyde in the reaction mixture and the second to the nominal amount of cationic Q-aldehyde in the reaction. The actual degrees of substitution were determined based on ^1^H-NMR data.

**Table 3:** Size and ζ-potential of chitosan and its synthesized derivatives.

№	Code	Hydrodynamic Radius, 0.5% AcOH, 5 mg/mL		SD	ζ-Potential (mV) 0.5% AcOH, 2.5 mg/mL
		Size, nm	Peak Area, %		
1	Chitosan	69.91	49	27.31	+16.8
		369.1	51	194.0	
2	H2.5	201.5	56	31.12	+29.2
		786.3	44	152.6	
3	H5.0	234.5	100	68.31	+27.1
4	H10	201.6	100	34.77	+29.0
5	H2.5Q6	70.04	5	13.17	+34.8
		369.8	95	107.9	
6	H2.5Q12	234.9	62	38.24	+32.0
		916.3	38	191.6	
7	H2.5Q25	201.9	41	40.76	+31.8
		677.1	59	198.1	
8	H5.0Q6	110.2	50	10.93	+38.1
		369.7	50	42.39	
9	H5.0Q12	149.2	31	27.72	+31.3
		317.9	69	96.82	
10	H5.0Q25	234.9	100	34.56	+32.3
11	H10Q6	369.7	100	49.75	+38.1
12	H10Q12	173.6	25	29.78	+37.7
		677.1	75	184.6	
13	H10Q25	51.75	24	8.396	+44.6
		787.7	76	179.8	

**Table 4:** Size and ζ-potential of synthesized chitosan derivatives.

№	Code	Hydrodynamic Radius, 0.01 M PBS, 5 mg/mL		SD	ζ-Potential (mV) 0.001 M PBS, 2.5 mg/mL
		Size, nm	Peak Area, %		
1	H2.5Q6	69.88	15	18.11	+21.5
		234.4	85	69.80	
2	H2.5Q12	149.2	100	24.27	+20.4
3	H2.5Q25	128.1	100	23.69	+17.1
4	H5.0Q6	24.30	9	4.586	+21.1
		149.3	91	45.13	
5	H5.0Q12	110.3	100	22.31	+17.7
6	H5.0Q25	13.25	4	1.073	+23.0
		128.1	96	34.55	
7	H10Q6	94.79	67	25.66	+25.9
		273.3	33	59.96	
8	H10Q12	60.22	15	9.974	+20.0
		173.6	85	58.88	
9	H10Q25	128.1	83	26.66	+24.9
		581.4	17	111.8	

## Data Availability

The original contributions presented in the study are included in the article. Further inquiries can be directed to the corresponding author.
